# Development of diagnostic SCAR markers for genomic DNA amplifications in breast carcinoma by DNA cloning of high-GC RAMP-PCR fragments

**DOI:** 10.18632/oncotarget.16704

**Published:** 2017-03-30

**Authors:** Shangyi Fu, Jingliang Cheng, Chunli Wei, Luquan Yang, Xiuli Xiao, Dianzheng Zhang, M. David Stewart, Junjiang Fu

**Affiliations:** ^1^ Key Laboratory of Epigenetics and Oncology, the Research Center for Preclinical Medicine, Southwest Medical University, Luzhou, Sichuan 646000, China; ^2^ Honors College, University of Houston, Houston, TX 77204, USA; ^3^ Department of Pathology, Affiliated Hospital of Southwest Medical University, Southwest Medical University, Luzhou, Sichuan 646000, China; ^4^ Department of Bio-Medical Sciences, Philadelphia College of Osteopathic Medicine, Philadelphia, PA 19131, USA; ^5^ Department of Biology & Biochemistry, University of Houston, Houston, TX 77204, USA; ^6^ Texas Heart Institute at St. Luke's Episcopal Hospital, Houston, TX 77030, USA; ^7^ Judicial Authentication Center, Southwest Medical University, Luzhou, Sichuan 646000, China

**Keywords:** high-GC primer, RAMP, random amplified polymorphic DNA (RAPD), sequence-characterized amplified region (SCAR), genomic instability

## Abstract

Cancer is genetically heterogeneous regarding to molecular genetic characteristics and pathogenic pathways. A wide spectrum of biomarkers, including DNA markers, is used in determining genomic instability, molecular subtype determination and disease prognosis, and estimating sensitivity to different drugs in clinical practice. In a previous study, we developed highly effective DNA markers using improved random amplified polymorphic DNA (RAPD) with high-GC primers, which is a valuable approach for the genetic authentication of medicinal plants. In this study, we applied this effective DNA marker technique to generate genetic fingerprints that detect genomic alterations in human breast cancer tissues and then developed sequence-characterized amplified region (SCAR) markers. Three SCAR markers (BC10-1, BC13-4 and BC31-2) had high levels of genomic DNA amplification in breast cancer. The *PHKG2* and *RNF40* genes are either overlapping or close to the sequences of SCAR marker BC13-4, while SCAR marker BC10-1 is in the intron and overlap the *DPEP1* gene, suggesting that alterations in the expression of these genes could contribute to cancer progression. Screening of breast cancer cell lines showed that the mRNA expression levels for the *PHKG2* and *DPEP1* were lower in non-tumorigenic mammary epithelial cell MCF10A, but elevated in other cell lines. The *DPEP1* mRNA level in invasive ductal carcinoma specimens was significantly higher than that of the adjacent normal tissues in women. Taken together, high-GC RAMP-PCR provides greater efficacy in measuring genomic DNA amplifications, deletion or copy number variations. Furthermore, SCAR markers BC10-1 and BC13-4 might be useful diagnostic markers for breast cancer carcinomas.

## INTRODUCTION

Breast cancer (BC) has the highest incidence of cancer occurrence in the female population and is the number one cause of cancer deaths for women worldwide [1–5]. In 2017, there were 252,710 estimated new cases of BC and 40,610 expected BC deaths among women in the United States [1]. Resource-stratified guidelines provide a vehicle for designing programs to promote early detection, diagnosis, and treatment using existing infrastructure and renewable resources and methods. Existing strategies for evaluating the current state and projecting future burden have a major role in developing new strategies to improve BC outcomes at the national and international levels [6–8]. While China's mainland currently has 470,000 BC patients, only 20 percent of them were diagnosed at early stage, whereas, in USA, 80 percent of BC patients were diagnosed at an early stage, securing best practice and best outcome for the patients (http://www.chinadaily.com.cn/china/2012-03/12/content_14814002.htm). Thus, we must increase BC risk awareness in the general public by recommending regular screening starting at 40 years of age to promote early detection and treatment.

Cancer is genetically heterogeneous regarding to molecular characteristics and pathogenic pathways. A wide spectrum of biomarkers, including DNA markers, is used for determining genomic instability, molecular subtype determination, and prognosis, and estimating sensitivity to different drugs in practice. Systematic analysis of genetic changes in colon cancer development demonstrated that multiple mutations are necessary for the evolution of normal cells into cancer cells [9]. There is growing evidence that multiple mutations are responsible for the development of cancer; cancer cells must exhibit mutator phenotypes [10, 11], which are likely to be responsible for the genomic instability found in cancer tissues. Mutator phenotype, including defective mismatch repair, is known to cause microsatellite instability, associated with multiple hereditary cancers [12, 13]. Loss of chromosomal materials would inactivate tumor suppressor genes while gain of chromosomal materials has the potential to activate tumor-promoting genes [8]. Genomic instability and chromosome copy number variations are the hallmarks of neoplastic transformation and a herald of genomic damage, leading to the conclusion that multiple mutations are the driving force in the carcinogenic processes [14–16]. Thus, the development of diagnostic markers for BC is vital for early detection and treatment of BC patients.

Since the 1990s, a number of genetic and DNA-based molecular marker techniques have been developed, including random amplified polymorphic DNA (RAPD) [17, 18]. Since then, RAPD technique either alone or in combination with other techniques is widely used for the genetic characterization of different medicinal plants, and other organisms [18, 19]. Although RAPD has few requirements of the amount of template DNA, produces no hazardous contamination, and is simple and inexpensive, the few disadvantages that poses including poor reproducibility and low production or yield. Resolution and production of RAPD are greatly increased by prolonging the RAMP time from the stage of annealing to extension in PCR [18, 19]. To improve the effectiveness of the RAPD method, we developed a highly effective DNA marker technique using improved RAPD with high-GC content primers, in which the GC content reaches 8–10 nucleotides for a 10 nucleotide primer (80~100%) [20, 21]. The Sequence Characterized Amplified Region (SCAR) markers are very stable, sensitive, and reliable. These markers are also effective for diagnostic purposes by using a simple PCR assay, which are generally derived from the molecular cloning of RAPD fragments in medicinal plants [21–28].

Different types of cancers have been associated with abnormal DNA fingerprinting; therefore, in this study we applied this effective improved RAPD technique to generate genetic fingerprints that detect genomic alterations in human breast cancer and develop diagnostic SCAR markers.

## RESULTS

### RAPD amplification from breast tumor tissue DNA samples using high-GC primers

**Figure 1:**
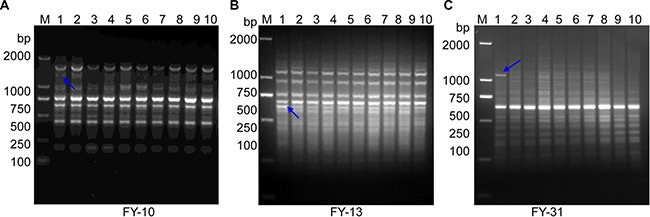
Improved RAPD PCR from breast cancer tissues and their adjacent tissues (**A**) High-GC primer FY-10. (**B**) High-GC primer FY-13. (**C**) High -GC primer FY-31. Five pairs of genomic DNA from breast cancer tissues and their adjacent or surrounding normal tissues were subjected to improved RAPD amplification (high-GC RAMP-PCR). Lanes 1, 3, 5, 7 and 9 are DNA from breast cancer tissues (see Table 1). Lanes 2, 4, 6, 8 and 10 are their matched DNA from adjacent tissue. The blue arrows indicate bands that were excised for DNA cloning. Lane “M” shows the DL2000 DNA molecular weight marker (bp).

To increase the efficiency of RAPD amplification and obtain more specific bands, standard primers were replaced with high-GC content primers (FY-10, FY-13 and FY-32). In addition, the RAMP time from the stage of annealing (36°C) to extension (72°C) was set to 5% (0.125 °C/s). This improved RAPD was used to amplify genomic DNA from five breast cancer patients. For each patient, two tissue samples were obtained from both the tumor and the normal tissue adjacent to the tumor. The PCR products were resolved by electrophoresis on a 1.5% agarose gel and showed signal difference in intensity of RAPD bands within patients (Figure 1).

### Cloning and sequencing of DNA fragments

**Figure 2:**
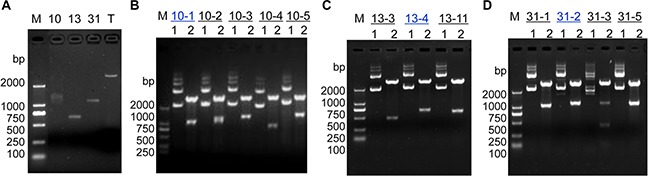
Molecular cloning for RAPD products (**A**) Agarose gel electrophoresis for RAPD DNA fragments 10, 13 and 31 derived from high-GC primers FY-10, FY-13 and FY-31, respectively. “T” indicates “pGM-T vector”. (**B**) Enzymatic identification of positive clones from RAPD fragment 10. (**C**) Enzymatic identification of positive clones from RAPD fragment 13. (**D**) Enzymatic identification of positive clones from RAPD fragment 31. Lane 1 contains undigested plasmid DNA; lane 2 contains plasmid DNA after E*coR*I digestion. Clones 10-1, 13-4 and 31-2 (blue text color) were selected for Sanger sequencing. Lane “M” contains the DL2000 DNA molecular weight marker (bp).

Three different bright bands with over-amplification in Figure 1 were excised and purified from the agarose gel for cloning into the pGM-T vector (Figure 2A). The positive cloned DNA fragments, 10-1, 13-4 and 31-2 (Figure 2B–2D) were selected based on insert sizes by E*coR*I digestion matching those of the RAPD fragments in Figure 1 and Figure 2A and then sequenced by the Sanger method. Sequence homology was determined using the online program BLAST (http://www.ncbi.nlm.nih.gov/BLAST/) against the GenBank database.

**Figure 3:**
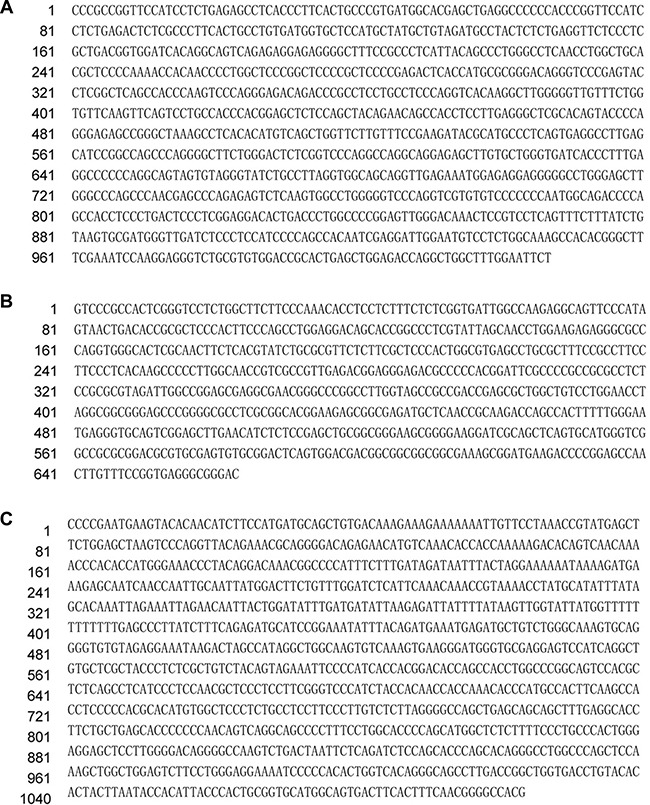
Results of Sanger-sequencing of the cloned DNA fragments (**A**) The sequence of clone 10-1. (**B**) The sequence of clone 13-4. (**C**) The sequences of clone 31-2.

**Figure 4:**
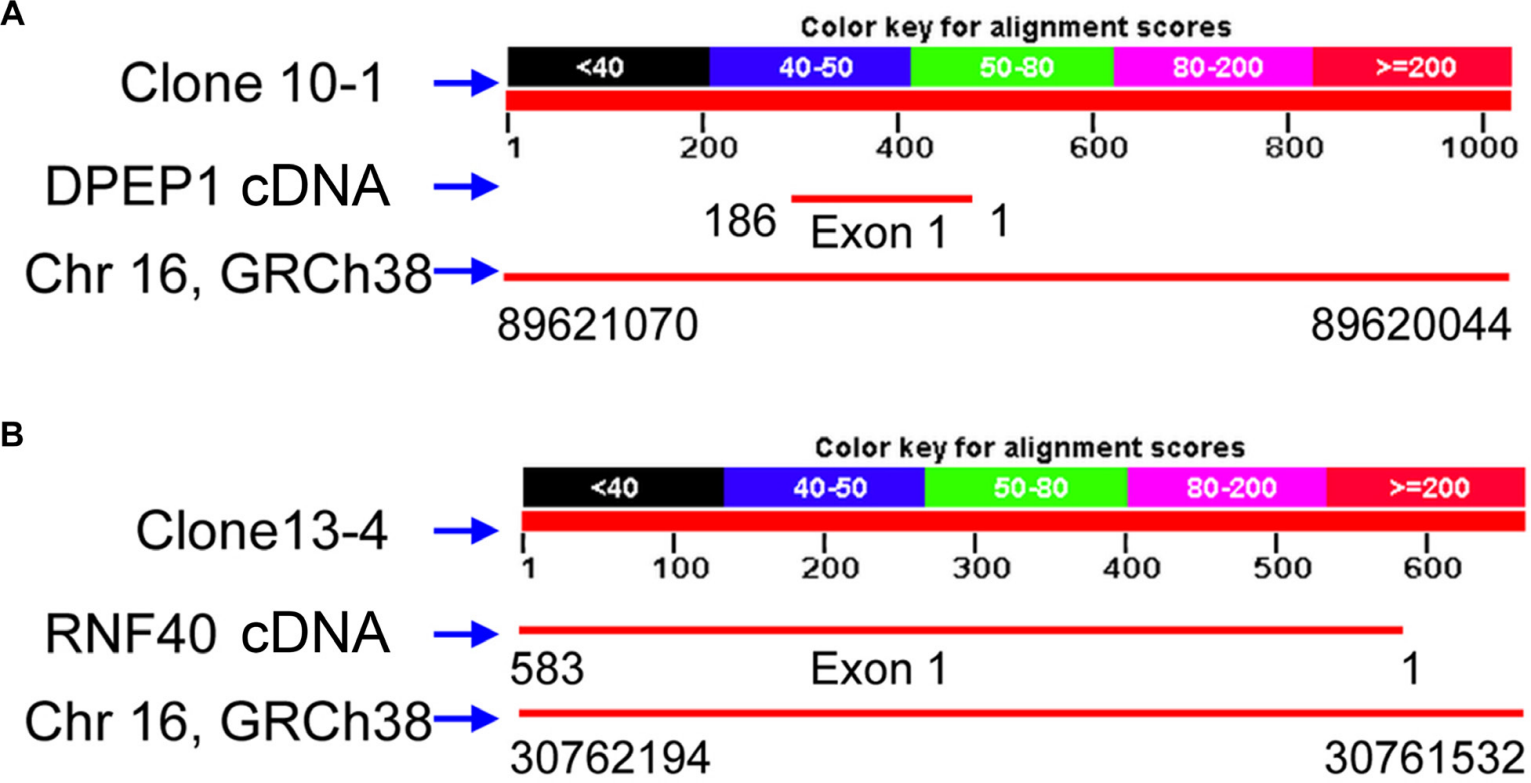
The Human genome locations of clones 10-1 and 13-4 with their partial cDNAs of DEPEP1 and RNF40, respectively

**Figure 5:**
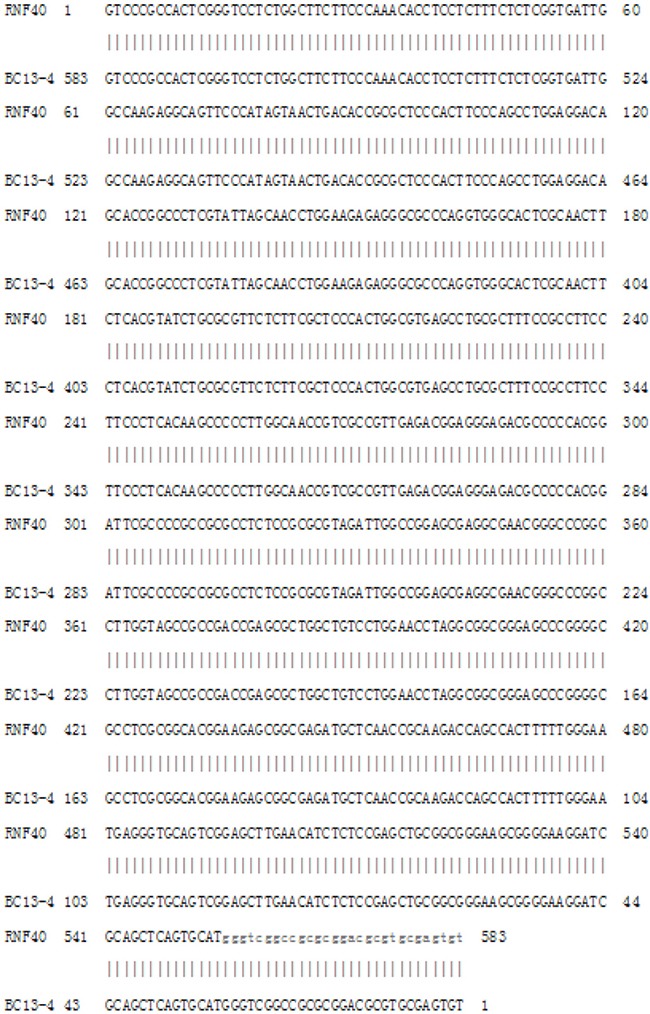
The sequence of clone 13-4 aligns with RNF40 cDNA Depicted is the BLAST output showing alignment of the clone 13-4 and *RNF40* cDNA sequences. The sequence of clone 13-4 showed 583bp (Plus strand) identity with the cDNA of *RNF40* (minus strand).

Sequencing of the aforementioned three cloned RAPD fragments from human breast cancer tissue revealed that clone 10-1 consisted of 1027 nucleotides is located in the intron of *DPEP1* transcript variant 2, and overlapped *DPEP1* (dipeptidase 1) transcript variant 1 in GenBank (NM_004413) (Figure 3A and Figure 4A). Clone 13-4 consisted of 663 nucleotides (Figure 3B), located only 536 bp away from the *PHKG2* gene (Homo sapiens phosphorylase kinase, gamma 2, NM_000294) (Homo sapiens chromosome 16, GRCh38 Primary Assembly. Sequence ID: ref|NC_000016.10|Length: 90338345, Range of clone13-4: 30761532 to 30762194 vs Range of PHKG2: 30748299 to 30761176) (Data not shown), and is also mainly located within or overlapping the first exon of *RNF40* (Homo sapiens ring finger protein 40, E3 ubiquitin protein ligase, NM_014771.3) (Figure 4B and Figure 5) in GenBank. Clone 31-2 consisted of 1104 nucleotides that did not match any known genes in GenBank (Figure 3C). Clone 10-1 is mapped to chromosome 16p24.3, clone 13-4 is mapped to chromosome 16p11.2, and clone 31-2 is mapped to chromosome 11q13.5.

### Development of diagnostic SCAR markers

**Figure 6:**
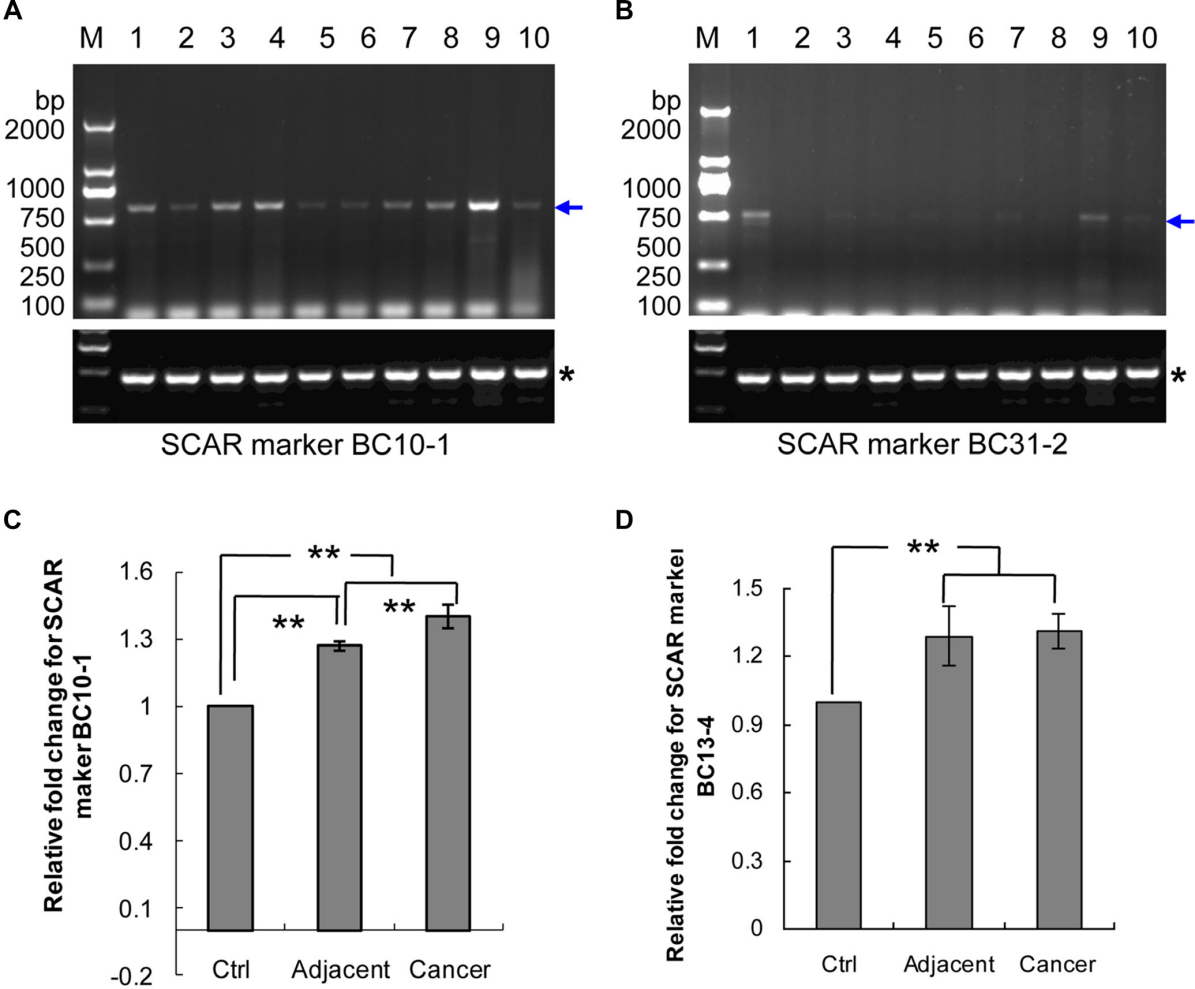
Genomic DNA amplification of SCAR markers BC10-1, BC13-4 and BC31-2 in breast cancer patients (**A**) SCAR marker BC10-1 in five pairs of genomic DNA from breast cancer tissues and their adjacent tissues. (**B**) SCAR marker BC31-2 in five pairs of genomic DNA from breast cancer tissues and their adjacent tissues. Lanes 1, 3, 5, 7 and 9 contain DNA from breast cancer tissues (see Table 1). Lanes 2, 4, 6, 8 and 10 contain their matched adjacent tissue DNA. Blue arrows indicate the amplified band, whereas the stars “*” indicate the internal control. (**C**) Real-time PCR for SCAR marker BC10-1. (**D**) Real-time PCR for SCAR marker BC13-4. “Cancer”, breast cancer tissues; “Adjacent”, normal tissues adjacent to or surrounding the breast tumor; “Ctrl”, normal women blood DNA; “**”*p* value ≤ 0.05.

To generate stable diagnostic SCAR markers from our cloned RAPD fragments, three pairs of primers for semi-quantitative PCR (Table 3) and three pairs of primers for real-time PCR (Table 4) were designed and synthesized based on the cloned sequences. The semi-quantitative SCAR primer pairs were used to amplify ten samples of genomic DNA collected from the breast tumors of five breast cancer patients. Genomic DNA from non-tumor adjacent tissue was used to test for amplification marker-specificity and to verify that the genomic DNA was over-amplified in the tumor. The PCR results indicated that the products with expected size were observed in all samples by three SCAR markers (Figure 6A, 6B, and data not shown). SCAR markers BC10-1 and BC31-2 showed higher signals indicating that these SCAR markers have genomic DNA over-amplified in the tumor tissues (Figure 6).

Real-time PCR was performed, including ten samples of women's blood DNA, 22 samples of adjacent normal tissue DNA and 30 samples of breast tumor tissue DNA. The results of SCAR marker BC10-1 showed gradually increased levels from blood DNA to tumor DNA (*p*-value ≤ 0.05) (Figure 6C). SCAR marker BC13-4 is present in increased levels in DNA from adjacent and tumor samples compared to blood DNA (*p*-value ≤ 0.05), but no difference in levels between adjacent DNA and tumor DNA (Figure 6D). SCAR marker BC31-2 did not show increased levels between blood DNA and tumor DNA (data not shown)

### *DPEP1* mRNA expression in BC cells and invasive ductal carcinomas

**Figure 7:**
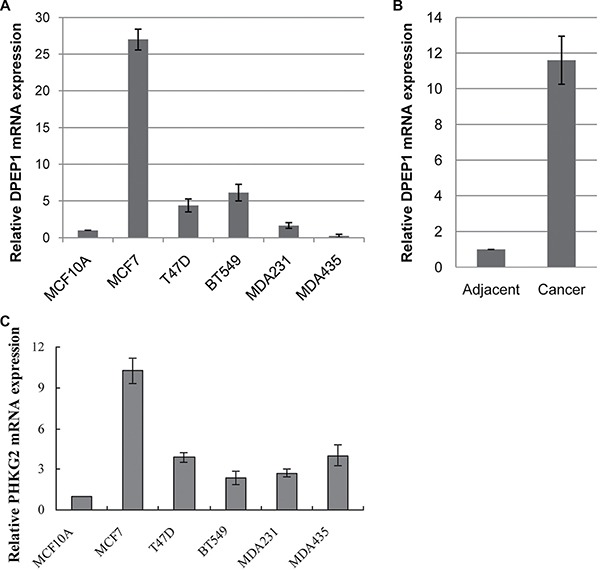
Analysis of DPEP1 and PHKG2 expression in breast cancer cell lines and tissues (**A**) *DPEP1* gene expression in breast cancer cell lines. (**B**) *DPEP1* gene expression in invasive ductal carcinoma specimens. (**C**) *PHKG2* gene expression in breast cancer cell lines. “MDA231”, MDA-MB-231 cells; “MDA435”, MDA-MB-435 cells.

The mRNA expression from the *DPEP1* gene was performed by real-time PCR using RNA extracted from BC cell lines BT549, MDA-MB-231 and MDA-MB-435, and non-tumorigenic mammary epithelial cell line MCF10A. We found that the level of *DPEP1* mRNA expression was lower in non-tumorigenic cell line MCF10A, but elevated in other BC cell lines (Figure 7A). To determine the *DPEP1* levels in BC development, we collected invasive ductal carcinoma specimens from 33 human BC patients and 11 adjacent normal tissues with informed consent. Total RNAs were purified from each tumor tissue and the surrounding normal tissues followed by real-time PCR. Figure 7B shows that the mRNA levels of *DPEP1* in BC tissues were significantly higher (11.6-fold) than that of the adjacent normal tissues.

### *PHKG2* mRNA expression in BC cells

The mRNA expression from the *PHKG2* gene was performed by real-time PCR using RNA extracted from breast cancer cell lines; we found that the level of *PHKG2* mRNA expression was lowest in the non-tumorigenic cell line MCF10A, but elevated in the other BC cell lines (Figure 7C).

## DISCUSSION

The RAPD technique either alone or in combination with other technique is widely used for the genetic characterization of different plants, animals or other organisms, particularly in medicinal plants [17–19]. In a recent cancer study, RAPD was also used for screening genome-wide changes in DNA methylation, which was called Methylation Sensitive-Random Amplified Polymorphic DNA-Polymerase Chain Reaction (MS-RAPD-PCR) [29]. Cancer patients are predisposed to fungal infections caused by *Candida albicans*, especially oral or respiratory tract candidiasis. Biernasiuk and colleagues [30] applied RAPD-PCR to successfully estimate genetic diversity of *C. albicans* isolated from the upper respiratory tract of patients with non-small cell lung cancer. However, there are some disadvantages to RAPD, like poor reproducibility and low yield of PCR products (low production). The resolution and production of RAPD are greatly increased by prolonging the RAMP time from the stage of annealing to extension in PCR [18, 19]. Recently, the technique was modified using high-GC content primers to improve the effectiveness of the RAPD method (high-GC RAMP-PCR technique) [20]. This improved RAPD successfully increased the number of RAPD bands produced from a given DNA sample. Using improved RAPD with high-GC content primers, we found that the average number of bands per primer increased from 6.41 to 15.04 in comparison to regular RAPD with standard primers. The average number of polymorphisms per primer was also increased from 4.647 to 10.22 [20]. Thus, we can produce more polymorphic bands using less RAPD primers. With these successes, we decided to apply this simple yet effective improved RAPD technique with high-GC primers to generate more fingerprints for detecting genomic alterations in human BC and developing diagnostic SCAR markers. High-GC primers FY-10, FY-13 and FY-32 were used to amplify genomic DNA successfully from five pairs of BC tissues and their adjacent tissues. Three different over-amplified bands were purified from the agarose gel, cloned and sequenced, and then used to successfully generate stable diagnostic SCAR markers BC10-1, BC13-4 and BC31-2.

Real-time PCR was performed using DNAs from bloods, tumor tissues and normal tissue adjacent to the tumor. SCAR marker BC10-1 results showed gradually increased levels from blood DNA to tumor DNA. SCAR marker BC13-4 are shown to have increased levels in both tumor tissues and normal tissues adjacent to the tumors compared to DNA from blood; however, there was no difference between DNA from tumor samples and adjacent tissues. SCAR marker BC31-2 did not show increased levels of tumor DNA compared to blood DNA. These results indicate that SCAR markers BC10-1 and BC13-4 could be useful diagnostic markers of genomic instability or chromosome copy number variation in BC [14–16].

To note, the *PHKG2* (Phosphorylase Kinase Subunit Gamma-2) gene, which encodes a subunit of phosphorylase kinase (*Phk*), is located close to SCAR marker BC13-4. Heritable deficiency of *Phk*, a regulatory enzyme of glycogen metabolism, is responsible for 25% of all cases of glycogen storage disease and occurs in 1 in 100,000 births. Liver phosphorylase b kinase (*PhK*) deficiency (glycogen storage disease type IX), one of the most common causes of glycogen storage disease, is brought by mutations in the *PHKA2*, *PHKB* and *PHKG2* genes. Mutations in the testis/liver isoform of *PHKG2*, which leads to approximately 10–15% of cases, have been associated with autosomal liver glycogenosis in gsd rats and humans [31–33]. However, the role of *PHKG2* in BC is unknown. Real-time PCR indicated that the level of mRNA expression for the *PHKG2* gene was lowest in non-tumorigenic BC cell line MCF10A, compared to elevated levels in all other tumorigenic BC cells tested. Of course, the levels of protein should be tested to further confirm their significance. Clone 10-1 was located and shown to overlap with *DPEP1* gene which promoted metastasis in colon cancer [34–36] but inhibited tumor cell invasiveness in pancreatic ductal adenocarcinoma [37]. The role of *DPEP1* in different types of cancer is controversial [34–38]. Our results, for the first time, showed that the mRNA levels of *DPEP1* in BC tissues were significantly higher (11.6-fold) than that of the adjacent normal tissues, which may play a role in promoting BC metastasis and invasiveness. Taken together, the *DPEP1* and *PHKG2* gene may be prognostic targets, which would play a vital role in BC carcinogenesis. Further studies of how the *DPEP1*, *PHKG2* and *RNF40* function in BC progression and how they might be used for risk prediction and therapy are underway.

## MATERIALS AND METHODS

### Breast carcinoma DNA and RNA preparation

Human breast invasive ductal carcinoma specimens were collected from surgically removed tumor tissues at Southwest Medical University Affiliated Hospital in China with informed consent [39–42]. The protocol for human samples was approved by the Ethics Committee of Southwest Medical University. The patients were Chinese women, between 26 to 68 years old and did not receive preoperative radiotherapy or chemotherapy. The histopathologic profiles of the 5 breast carcinomas specimens for RAPD amplification are shown in Table 1. The cancer tissues and adjacent tissues or surrounding normal tissues were immediately frozen in liquid nitrogen and stored at −80°C. DNA extraction from tissues was performed using the Proteinase K and phenol/chloroform method [40, 43–45]. Total RNA was isolated from cancer cells and tissues using the RNeasy Mini Kit (50) (Cat No.74104, QIAGEN) and stored at −80°C. The RNA and DNA quality was determined by a 1% agarose gel electrophoresis and spectrophotometry. Genomic DNA from healthy women was also isolated from peripheral leukocytes or blood using the previously described method [43]. The final concentration of all DNA samples was adjusted to 20 ng/μl and stored at −20 °C until use.

**Table 1:** The histopathologic profiles of the 5 breast carcinomas specimens

	1 (TG53)	3 (TG55)	5 (TG57)	7 (TG59)	9 (TG62)
Histology	Ductal	Ductal	Ductal	Ductal	Ductal
Diagnostic age	49	38	68	45	48
Stage	T3	T4	T2	T2	T3
Grade	II	III	II	III	III
Estrogen receptor	+,90%	−	−	−	−
Progesterone receptor	+,10%	−	−	−	−
HER-2	1+	1+	0	3+	0
E-Cadherin	+	+	+	+	+
p53	−	+, 90%	+, 40%	+, 30%	+, 60%
Ki-67	+, 40%	+, 75%	+, 70%	+, 15%	+, 50%

### High-GC primers for RAPD

The 10-bp length oligos with high-GC contents (90% of G+C) were designed and synthesized at Beijing DNA chem. Biotechnology Co., Ltd (Beijing, China) and were as previously described [20]. The sequences of high-GC primers FY-10, FY-13 and FY-31 are presented in Table 2.

**Table 2:** The sequences of high-GC primers

Name	Sequence (5′-3′)
FY-10	GCTCCCGCCG
FY-13	GCGTCCCGCC
FY-31	CGTGGCCCCG

### Amplification of DNA by high-GC RAPD PCR

All the contents of the PCR (10 μl total) were as follows: 1 μl of 2.5 μmol/L primers, 0.5 μl of DNA template (10 ng), 5 μl of 2×PCR Taq MasterMix (TianGen Biotech Co. Ltd., Beijing, China), 3.5 μl of deionized water. PCR conditions were as follows: initial denaturation at 95°C for 90 s, followed by 40 cycles of 40 s at 94°C, 60 s at 36°C, 90 s at 72 °C, and final extension of 5 min at 72°C. PCR amplification was executed in an “Applied Biosystems Veriti^®^ 96-Well Thermal Cycler” (Life Technology, USA), and the RAMP time from annealing to extension with a RAMP rate for 5% (0.125 °C/s) was used [20, 21].

### Agarose gel electrophoresis

The amplified RAPD-PCR products were resolved by electrophoresis on a 1.5% agarose gel in 1× TAE (Tris-acetate-EDTA) buffer at 60 volts for 210 minutes at 4 °C. To increase the resolution, the electrophoresis time was extended to 240 minutes at 60 volts at 4 °C. Then, the gels were visualized by 0.5 μg/mL ethidium bromide (EB) staining, and the images were documented using the ChemiDoc XR (Bio-Rad, USA) [19, 43]. Bands with different intensity between BC tumors and adjacent normal tissues were selected for cloning.

### Molecular cloning of RAPD fragments from amplification by high-GC primers

Cloning of RAPD fragments was described previously [21, 23, 27, 28, 44]. Bands with differential amplification were excised from agarose gels and purified with a TIANgel Mini Purification Kit (DP209, China) according to the company provided protocol. Purified DNA fragments were ligated into pGM-T vector (VT202, Tiangen reagents, Beijing, China), and transformed into DH5α *E. coli* complement cells. The recombinant clones were selected on LB agar plates from blue and white colonies. The presence of right insert was verified by PCR by using T7/SP6 primer pairs, and *EcoR*I digestion from purified plasmids. The positive clones were selected for Sanger sequencing.

### Development of diagnostic SCAR markers

Homology searches within the human genome were performed using BLAST (http://www.ncbi.nlm.nih.gov/BLAST/). The sequence of each cloned RAPD fragment was used to design pairs of SCAR primers using Primer 3 software (http://frodo.wi.mit.edu/primer3). Sequences of the SCAR primers, amplification length and PCR conditions are shown in Table 3. Ten DNA samples, which we mentioned previously, were used as templates for PCR amplification for development of SCAR markers. The PCR reaction solution consisted of 5 μl 2×Taq PCR MasterMix, 1 μl of 2.5 μM each pair of SCAR primers and 1 μl of genomic DNA (20ng), with a total volume of 10μl. Amplification reactions were performed with an initial pre-denaturation of 90 s at 95°C followed by 33 cycles of denaturation at 94°C for 40 s, annealing at 60°C for 30 s, and extension at 72°C for 30 s. The final extension step was performed at 72°C for 5 min. The amplified PCR products were resolved by electrophoresis on a 1.5% agarose gel [19, 43]. For semi-quantitative genomic DNA PCR using SCAR markers, *GAPDH* gene served as a loading control.

### Quantitative real-time PCR amplification

Three pairs of primers for real-time PCR of SCAR markers and *β-actin* were designed using software from assay design center of Roch (http://lifescience.roche.com/shop/CategoryDisplay?catalogId=10001&tab=&identifier=Universal+Probe+Library). Sequences of real-time PCR primers and probes for genomic DNA are shown in Table 4. Real-time PCR was performed using the *FastStartUniversal* *Probe* Master *(Rox)* (Cat No. 4914058001, Roch) and the StepOnePlus Real-Time PCR system (Applied Biosystems, USA) as described previously [39, 41, 42, 46–48]. The *β-actin* gene served as an internal control for normalizing the relative levels of SCAR markers.

**Table 3:** Sequences of SCAR primers, PCR product size (bp)

SCAR	5′-primer	Sequence (5′-3′)	3′-primer	Sequence (5′-3′)	Size	Tm (°C)
BC10-1	BC10-1L	GATCACAGGCAGTCAGAGAGG	BC10-1R	CAGGCCACTTGAGACTCTCTG	590	60
BC31-2	BC31-2L	ACAAACGGCCCCATTTCTTTG	BC31-2R	TTGGTGGTTGTGGTAGATGGG	512	60
GAPDH	GAPDH5G	ACCCAGAAGACTGTGGATGG	GAPDH3G	TGACAAAGTGGTCGTTGAGG	376	60

Abbreviations: SACR, sequence-characterized amplified region; bp, base pair.

**Table 4:** Sequences of real time PCR primers for SACR markers, probes

SCAR	5′-primer	Sequence (5′-3′)	3′-primer	Sequence (5′-3′)	Probe
BC10-1	BC10-1L87	cccaggcagtagtgtagggta	BC10-1R87	ccctcctctccatttctcaa	87
BC13-4	BC13-4L34	tctggcttcttcccaaacac	BC13-4R34	cgcggtgtcagttactatgg	34
beta-actin	Q-b-actin55LG	aagtcccttgccatcctaaaa	Q-b-actin55RG	atgctatcacctcccctgtg	55

Abbreviations: SACR, sequence-characterized amplified region.

### Real-time PCR analysis of *PHKG2* and *DPEP1* mRNA expression

The cDNA libraries were generated from 1 μg of total RNA using a reverse transcriptase kit (Invitrogen, USA). The method for real-time PCR was described previously [41, 42, 46–48]. Sequences of the primers and probes for human *PHKG2* and *DPEP1* mRNA are shown in Table 5. Levels of the 18S rRNA were measured in the same reaction for each sample to serve as an internal control for normalizing the relative mRNA levels of *PHKG2* and *DPEP1*.

**Table 5:** Sequences of real time PCR primers for PHKG2 and DPEP1 mRNA expression, probes

Gene	5′-primer	Sequence (5′-3′)	3′-primer	Sequence (5′-3′)	Probe
PHKG2	Q-PHKG2-L2	caatatgcagatccgactttca	Q-PHKG2-R2	ggggtcccacacaactctc	2
DPEP1	Q-DPEP1-13L	tgcactgcagacttctttcg	Q-DPEP1-13R	gccaggggaggtcattgt	13
18S	18S48R	gcaattattccccatgaacg	18S48L	gggacttaatcaacgcaagc	48

### Statistical analysis

Student's *t*-test was used to determine any significant differences. A *p*-value of less than 0.05 was considered to be significant for all tests. All the experiments were repeated thrice.
